# Phase Transformations Caused by Heat Treatment and High-Pressure Torsion in TiZrHfMoCrCo Alloy

**DOI:** 10.3390/ma16041354

**Published:** 2023-02-05

**Authors:** Alena S. Gornakova, Boris B. Straumal, Alexander I. Tyurin, Natalia S. Afonikova, Alexander V. Druzhinin, Gregory S. Davdian, Askar R. Kilmametov

**Affiliations:** 1 Osipyan Institute of Solid State Physics of the Russian Academy of Sciences, Ac. Osipyan Str. 2, 142432 Chernogolovka, Russia; 2 G.R. Derzhavin Research Institute “Nanotechnologies and Nanomaterials” TSU, Internazionalnaja Str. 30, 392000 Tambov, Russia

**Keywords:** high-entropy alloys, high-pressure torsion, Laves phases, solid solutions, heat treatment, nanohardness, Young’s modulus

## Abstract

In this work the high-entropy alloy studied contained six components, Ti/Zr/Hf/Mo/Cr/Co, and three phases, namely one phase with body-centered cubic lattice (BCC) and two Laves phases C14 and C15. A series of annealings in the temperature range from 600 to 1000 °C demonstrated not only a change in the microstructure of the TiZrHfMoCrCo alloy, but also the modification of phase composition. After annealing at 1000 °C the BCC phase almost fully disappeared. The annealing at 600 and 800 °C leads to the formation of new Laves phases. After high-pressure torsion (HPT) of the as-cast TiZrHfMoCrCo alloy, the grains become very small, the BCC phase prevails, and C14 Laves phase completely disappears. This state is similar to the state after annealing at high effective temperature *T*_eff_. The additional annealing at 1000 °C after HPT returns the phase composition back to the state similar to that of the as-cast alloy after annealing at 1000 °C. At 1000 °C the BCC phase completely wets the C15/C15 grain boundaries (GBs). At 600 and 800 °C the GB wetting is incomplete. The big spread of nanohardness and Young’s modulus for the BCC phase and (C15 + C14) Laves phases is observed.

## 1. Introduction

The so-called high-entropy alloys (HEAs) are known also as multiprincipal alloys, baseless alloys, or alloys without a main component. For the first time, HEAs were studied by Brian Cantor [1] and Jian-Wei Ye [2] with co-authors. The first HEAs were alloys with six or more components in equiatomic proportions. The big surprise for the researchers was that such alloys can form a homogeneous disordered solid solution. It turned out that this is due to the fact that the unique combination of atoms with different atomic radii in HEAs can greatly hinder the nucleation of the second phases and their subsequent growth [1,2,3,4,5]. Such alloys may have better properties than traditional alloys. In particular, they have high hardness [3], good strength at high temperatures [4], and excellent resistance to wear, oxidation, and corrosion [5]. HEAs are promising materials in many fields from medicine to the nuclear industry. Due to the fact that HEAs exhibit promising mechanical, magnetic properties and corrosion resistance, this combination of properties makes them potential candidates for components of next-generation nuclear reactors and other environments with high levels of radiation [6,7,8]. The second area of HEAs development is implants in medicine, as an analogue of replacing existing imperfect titanium ones. The development of a new generation of metallic biomaterials with both biocompatibility and good mechanical properties is necessary to meet the needs of future medical applications. The TiZrHfMoCrCo alloy studied in this work is one of the newly developed alloys, it belongs to the new class of structural and functional HEA materials [9]. It is important for HEAs used in medicine as implants that all constituent elements of such HEAs are biocompatible. Co–Cr and Co-Cr–Mo alloys are generally accepted metallic biomaterials [10,11,12,13,14,15], as well as titanium-based alloys, especially for surgical implants and dental alloys. They are also widely used as sliding materials in artificial joints [16]. The TiZrHfMoCrCo alloy studied in this paper belongs to promising biomaterials, its biocompatibility is comparable to commercially pure titanium, which is used for orthopedic implants. Detailed reviews on HEAs can be found in References [17,18,19].

The most commonly used approaches to the manufacture of HEAs are casting or arc melting technologies [1,2,3,4,5,6,7,8]. In this work, the HEA was obtained by levitation melting in an atmosphere of pure argon. This technique allows to obtain well-mixed ingots of high purity. Thus, the use of two new methods, such as the method of levitation melting in the argon atmosphere and one of the methods of severe plastic deformation, namely the high-pressure torsion (HPT), provided us the opportunity to obtain new data on the microstructure and phase transformations in the TiZrHfMoCrCo alloy. The purpose of our work was to study the effect of heat treatment and HPT on the microstructure and phase transformations in the TiZrHfMoCrCo alloy, as well as to measure the hardness and Young’s modulus of its structural components.

## 2. Materials and Methods

The high-entropy alloy Ti10.84Zr18.73Hf42.34Mo20.77Cr3.82Co3.50 (wt.%) or Ti22.14Zr20.26Hf23.37Mo21.43Cr7.17Co5.64 (at.%) (subsequently referred to as TiZrHfMoCrCo) was smelted by levitation melting in an argon atmosphere in the form of a cylindrical ingot with a diameter of 10 mm. Pure metals were used for the preparation of the alloy, namely titanium grade Ti-I (iodide titanium 99.98 wt.%), zirconium (iodide zirconium 99.98 wt.%), molybdenum (99.97 wt.%), hafnium (iodide hafnium 99.95 wt.%), cobalt (99.9 wt.%), and chromium (99.99 wt.%). The analysis of the ingots showed its structural and chemical uniformity in cross-section and length. Component analysis was performed on a FEI dual beam Versa 3D HighVac scanning electron microscope (SEM) manufactured by FEI (Hillsboro, OR, USA). The 0.8 mm thick disks were cut off from the ingot by the spark erosion. After removing the surface riveted layer with grinding paper, the samples were annealed in evacuated quartz ampoules, the initial residual pressure in which was about 8 × 10^−3^ Pa. Annealing of the disks was carried out at temperatures of 1000 °C (24 and 336 h), 800 °C (336 h), and 600 °C (480 h). Two disks of the initial alloy were subjected to HPT at room temperature (5 revolutions of the plunger under pressure of 7 GPa at a rotation speed of 1 revolution per minute) in a Bridgman anvil-type installation using computer control manufactured by W. Klement GmbH, Lang, Austria. After the HPT, the thickness of the samples was 0.3 mm. One of the samples after the HPT was annealed at 1000 °C, for 96 h. Measurements of the nanohardness and Young’s modulus of the initial sample were carried out on the TI-950 Triboindenter equipped with a Berkovich indenter. Measurements were carried out in different areas of the sample which appeared light and dark in the SEM micrographs, as well as along the diameter of a disk, the loading rate was constant and equal to *dP*/*dt* = 40 mN/s. Before the measurements, the surface of the samples was polished on a diamond paste with a grain size of 1 μm. Numerical values of hardness (*H*) and Young’s modulus (*E*) of the studied samples were determined using the Oliver–Pharr method based on characteristic *P*-*h* diagrams [13,14,15]. X-ray diffraction patterns were obtained using a Siemens D-500 X-ray diffractometer in Cu-Kα radiation and used for structural phase analysis of samples. The phase analysis and calculation of the lattice parameters were carried out using the PowderCell 2.4 program (PowderCell for Windows, Version 2.4. 08.03.2000, Werner Kraus and Gert Nolze, BAM Berlin, Germany).

## 3. Results

**Figure 1:**
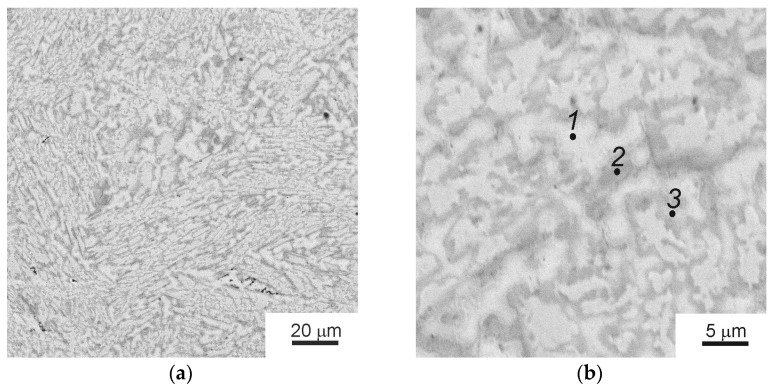
SEM micrographs of the as-cast TiZrHfMoCrCo alloy: (**a**) general view, (**b**) the component analysis areas are marked with dots: light gray (*1*), dark gray (*2*), and gray (*3*) areas.

Figure 1a shows a low-magnification SEM micrograph of the as-cast initial TiZrHfMoCrCo alloy. The high-magnification micrograph in Figure 1b shows three kinds of areas appearing light gray (*1*), dark gray (*2*), and gray (*3*).

**Figure 2:**
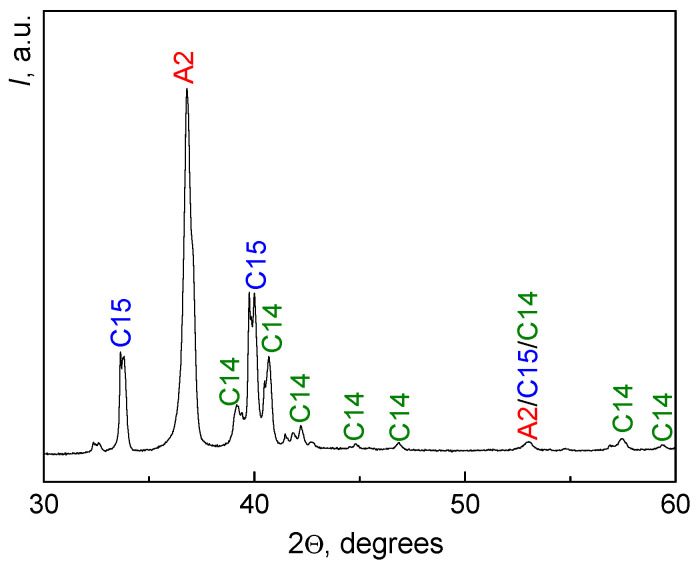
Diffraction pattern of the initial as-cast TiZrHfMoCrCo alloy.

Table 1 contains data on composition of areas *1, 2,* and *3*. Hafnium is present uniformly in all areas, the most significant difference in all areas concerns molybdenum content. The regions (*1*) have the lowest Mo concentration (7 wt.%), and regions (*3*) have the highest Mo concentration (34 wt.%). The results of X-ray analysis are shown in Figure 2. The figure shows the peaks of A2, C14, and C15 phases. The designation “A2/C15/C14” corresponds to the overlap of peaks from all three phases. In Figure 2, splitting can be seen at the peaks of intensities related to the Laves phases (C15 and C14). We suppose that this splitting is due to the resolution of the doublet at small angles because of the peculiarities of the macrostructure of the Laves phase.

The initial as-cast alloy contains three main phases, namely the (A2) phase with body-centered cubic (BCC) lattice (Ti,Zr,Hf)cub Im3m, the C14 Laves phase Cr_2_Zr P63/mmc, and the C15 Laves phase (Mo,Cr)_2_Zr Fd3m. The ratio of the fractions of these phases in the alloy is 51:23:26. Correlating the X-ray diffraction analysis data and the SEM data, it was found that the BCC phase A2 corresponds to the composition of the light-gray areas (*1*), the phase C15 corresponds to the composition of dark-gray areas (*2*), and the phase C14 corresponds to the composition of the gray areas (*3*).

**Figure 3:**
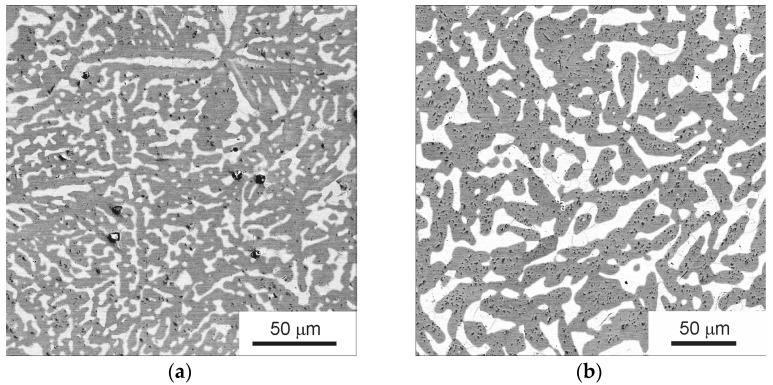
SEM images of the microstructure of the TiZrHfMoCrCo alloy annealed at 1000 °C: (**a**) 24 h and (**b**) 336 h.

Two annealings were carried out at a temperature of 1000 °C for 24 h (Figure 3a) and 336 h (Figure 3b). The effect of temperature and duration of annealing on the microstructure and phase composition of the TiZrHfMoCrCo alloy was investigated. It can be seen that the microstructural components are enlarged and the phase ratio changes (Table 2).

**Figure 4:**
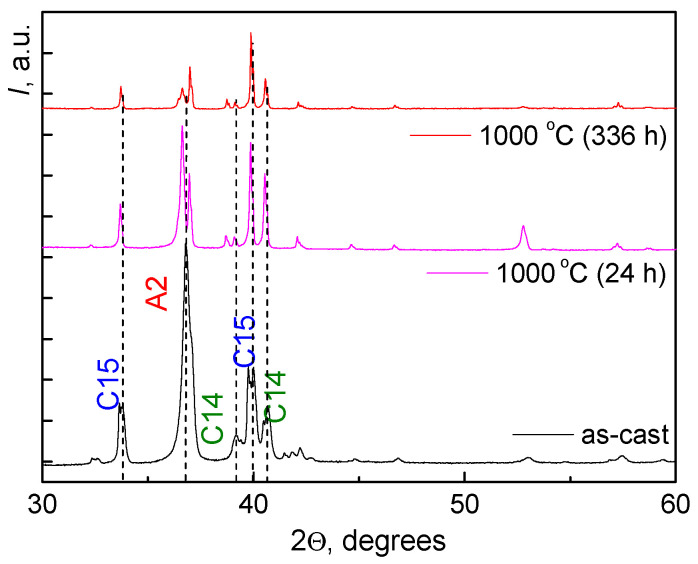
Diffraction patterns of the TiZrHfMoCrCo alloy in the as-cast state (black line) and after annealing (red line: 1000 °C for 336 h; and pink line: 1000 °C for 24 h).

X-ray diffraction analysis showed that annealing at 1000 °C leads to a change in the percentage of phases as well as the change in the lattice parameters of the phases (Figure 4). After annealing, the BCC A2 phase almost disappeared. The lattice parameter in the remaining BCC A2 phase increased from *a* = 0.3448 to *a* = 0.3460 nm. The percentage of C15 Laves phase increased from 26% to 86%. Its lattice parameter decreased from *a* = 0.7501 nm to *a* = 0.7473 nm. The percentage of C14 Laves phase decreased from 23% to 10%. Its lattice parameters remained almost constant.

**Figure 5:**
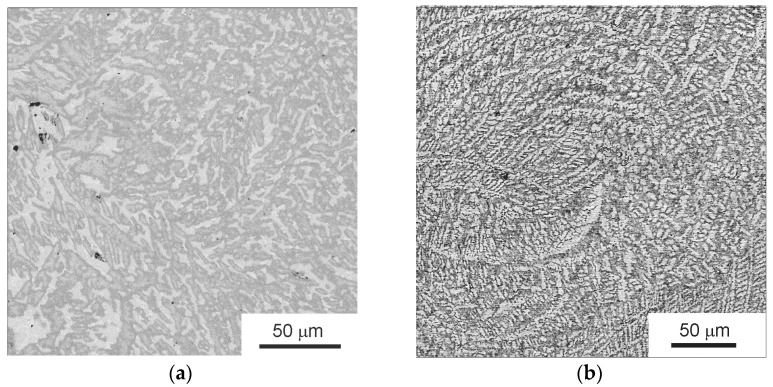
SEM images of the microstructure of the TiZrHfMoCrCo alloy annealed at: (**a**) 800 °C for 336 h and (**b**) 600 °C for 480 h.

**Figure 6:**
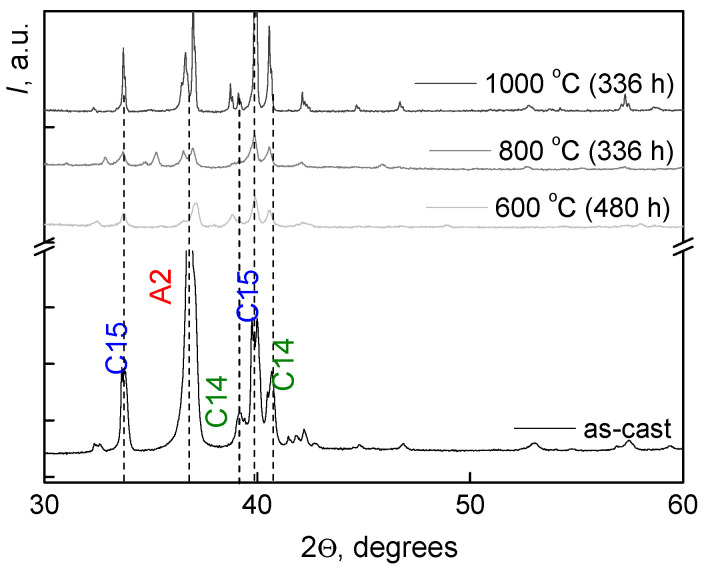
Diffraction patterns for the TiZrHfMoCrCo alloy in the as-cast state (black line) and annealed state (light gray: 600 °C for 480 h; gray: 800 °C for 336 h; and dark gray: 1000 °C for 336 h).

Figure 5 shows the SEM images of the microstructure of the TiZrHfMoCrCo alloy annealed 800 °C for 336 h (a) and 600 °C for 480 h (b). Figure 6 contains the diffraction patterns for annealed samples. The peaks corresponding to the three main phases are marked.

The anneals at 800 and 600 °C (Figure 5) showed that the (A2) phase is stable in the temperature range of 600–1000 °C (Table 3), its percentage is low (around 4 %) and lattice parameter remains almost constant.

The fraction of Laves phase C15 decreases from 86 to 60% with a decrease in the annealing temperature. Its lattice parameter increases from 0.7473 to 0.7498 nm. The fraction of Laves phase C14 increases from 10 to 27% with a decrease in the annealing temperature. Its lattice parameter remains almost constant. However, a kind of “structural stratification” occurs in the Laves phase C14 at 600 °C. The C14 phase splits into two structural types, C14 and C14-2, with different lattice parameters. Their percentage is 10 and 7%, respectively. According to the results of X-ray diffraction analysis at 600 °C, another (C36) phase was detected, with 9%. The last line “Additional phases” in Table 3 includes a small amount of oxides, carbides, and nitrides.

**Figure 7:**
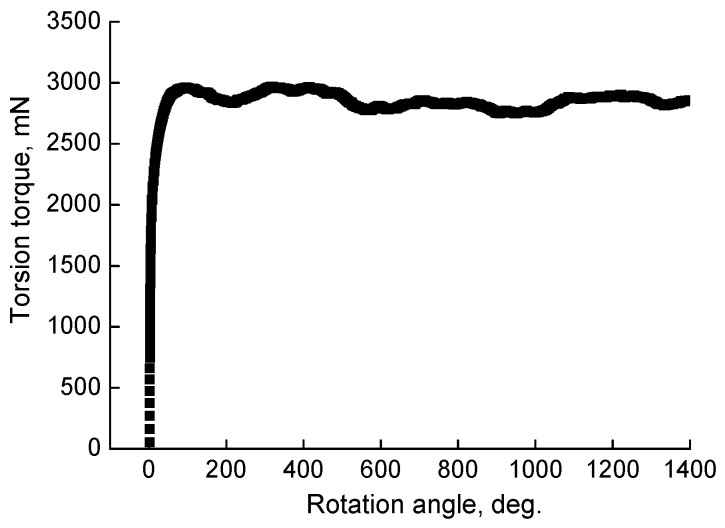
HPT loading curve for the as-cast TiZrHfMoCrCo alloy.

HPT, similar to heat treatment, has a significant effect on the structure and phase composition of the TiZrHfMoCrCo alloy. Figure 7 shows the loading curve (dependence of torsion torque on the plunger rotation angle) for the initial alloy. The HPT leads to the disappearance of the phase (C14) and provokes the appearance of another solid solution based on molybdenum (Mo)cub (A2-2) (Table 4). Annealing after the HPT changes the phase composition: phase (A2-2) disappears, and phase (C14) appears again.

**Figure 8:**
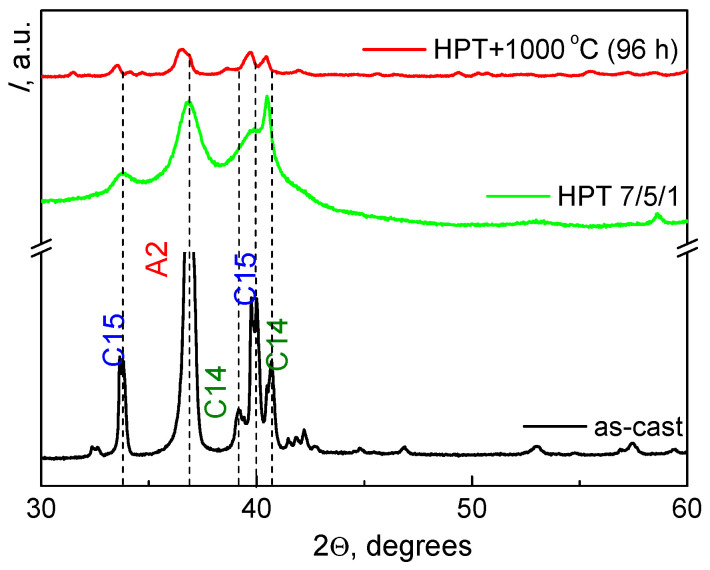
Diffraction patterns for the TiZrHfMoCrCo alloy in the as-cast state (black line) after HPT (green line) and after HPT plus annealing at 1000 °C 96 h (red line).

**Figure 9:**
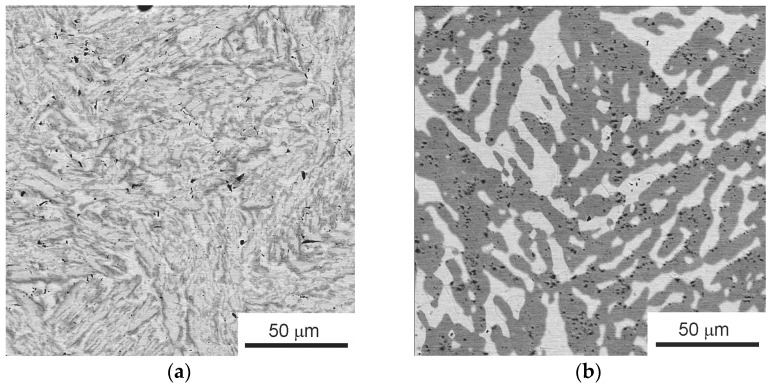
SEM images of the microstructure of the TiZrHfMoCrCo alloy (**a**) the as-cast sample subjected to HPT, (**b**) HPT plus additional annealing at 1000 °C 96 h.

The amount of phase A2 is significantly reduced after annealing at 1000 °C, compared with the initial state and state after HPT, and the Laves phases prevail again (C15) + (C14) = 80% like after annealing the as-cast alloy (Figure 8). The combination of HPT and annealing at 1000 °C provides about 5% of additional phases. We believe that the appearance of additional phases (Table 3 and Table 4) is due to the annealing in quartz ampoules, which does not provide a good vacuum. HPT does not lead to uniform grinding of phases (Figure 9a) and it is difficult to find a significant difference from the original structure (Figure 1a). The annealing after HPT leads (as can be expected) to the grain growth in constituent phases (Figure 9b).

**Figure 10:**
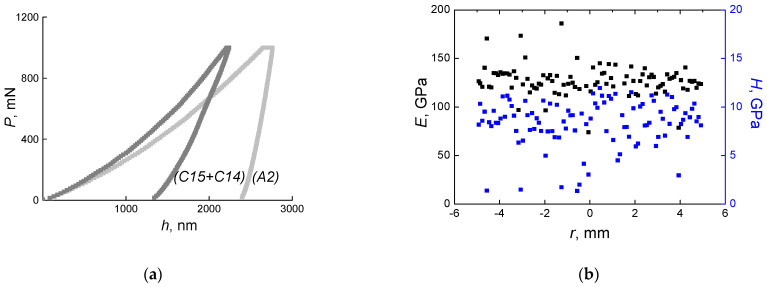
(**a**) *P*-*h* diagrams for the TiZrHfMoCrCo alloy taken from areas (A2) and (C15 + C14). (**b**) Dependences of nanohardness (*H*) and Young’s modulus (*E*) for the TiZrHfMoCrCo alloy taken from the sample diameter.

In this work, the values of nanohardness (*H*) and Young’s modulus (*E*) of individual phases (A2) and (C15 + C14) in the initial alloy were measured. For (C15 + C14) phase *H* = 9.5 ± 1.0 GPa, and *E* = 128 ± 6 GPa; and for (A2) phase *H* = 4.9 ± 0.2 GPa, and *E* = 232 ± 7 GPa. Figure 10a shows *P*-*h* diagrams, it is clearly visible that the dark (C15 + C14) phase is harder. Having measured *H* and *E* along the diameter of the sample (Figure 10b), we obtained a large data spread from 7 to 12 GPa for *H*, and from 120 to 150 GPa for *E*. At each measurement, we fall either into the grain of one or another phase, or into the interphase boundaries.

## 4. Discussion

An important direction for improving the properties of high-entropy alloys is the transition from single-phase HEAs to two- and multi-phase materials. Let us consider the case of HEAs including titanium, zirconium, and hafnium, as in this work. Titanium, zirconium, and hafnium are located one below the other in the fourth subgroup of Mendeleev’s periodic system of elements. Therefore, all three of these elements at high temperatures have a BCC lattice with the A2 structure, space group Im3m (β-Ti, β-Zr, β-Hf), and at low temperatures, a hexagonal close-packed (hcp) lattice with the A3 structure, space group C6mmc (α-Ti, α-Zr, α-Hf). The transition between BCC and hcp lattices occurs at temperatures of 882 °C in titanium, 863 °C in zirconium, and 1743 °C in hafnium. In binary phase diagrams, there are regions of infinite mutual solubility between these three elements above and below the α-β transformation. A narrow two-phase region also appears in these diagrams where the α and β phases are in equilibrium. The temperature width of this region does not exceed ~60 °C. The same applies to the Ti-Zr-Hf ternary diagram. Above the α-β transformation temperature, there is an area of infinite solubility based on the β-phase, and below this temperature, an area of infinite solubility based on the α-phase.

Elements such as niobium, tantalum, and molybdenum have a BCC lattice from 0 K to their melting point. They also form an infinite series of solid solutions with the high-temperature β-phase of titanium, zirconium, and hafnium. Therefore, it is not surprising that five- or six-component Ti-Zr-Hf-Ta-Nb-Mo HEAs consist of a high temperature at BCC phase A2. The second phase does not appear in these HEAs even if their composition strongly deviates from the equimolar one. Alloys of the Ti-Zr-Hf-Ta-Nb system begin to solidify at a temperature of about 2000 °C. Such HEAs become two-phase only when the temperature drops significantly below the solidification temperature. Therefore, during rapid cooling, low-temperature phases do not have time to form, and in the cast state Ti-Zr-Hf-Ta-Nb alloys consist of only one BCC phase [20,21,22,23,24,25,26,27]. Calphad calculations show that the lower limit of the existence of a high-temperature β-phase in the Ti-Zr-Hf-Ta-Nb-Mo HEA can start at about 1100 °C [9,22,26]. Below this temperature, not only hcp phases with the A3 structure can appear, but also other BCC phases A2 with a different lattice period, as well as various Laves phases [9,22,26]. For example, the equimolar Ti-Zr-Hf-Ta-Nb alloy contains a single BCC phase A2 not only in the as-cast state, but also after annealing at temperatures of 1200 and 1000 °C [20]. However, if this alloy is annealed at a temperature of 800 °C, then the second BCC phase A2 appears with a smaller lattice period.

The situation becomes more interesting if we add to titanium, zirconium, and hafnium not only metals with a BCC lattice such as Ta, Nb, and Mo, but also the so-called beta stabilizers. These include, for example, chromium, cobalt, copper, or nickel. These elements are called beta stabilizers since when they are added to titanium, zirconium, or hafnium, the temperature of the α-β transformation decreases significantly. On the other hand, the solubility of beta stabilizers in titanium, zirconium, and hafnium is limited. Therefore, HEAs with the addition of these elements become two-phase not only with decreasing temperature, but also with a significant deviation of the composition from equimolar one [9,22,23,28,29,30,31,32].

The TiZrHfMoCrCo alloys studied in this work, belong to the HEAs with beta stabilizers. In addition to titanium, zirconium, and hafnium, it contains BCC metal molybdenum, as well as beta stabilizers chromium and cobalt. Therefore, already in the as-cast state, only approximately a half of it is the BCC phase. The other half is equally divided between the Laves phases C15 and C14. A similar phase composition with a predominance of the BCC phase and small additions of Laves phases was also observed in TiZrHfCo0.07Cr0.07Mo HEA of similar composition [9]. Annealing at 1000 °C leads to the almost complete disappearance of the BCC phase A2 (see Table 2). At the same time, the amount of the C15 Laves phase rises strongly to 86%, while the proportion of the C14 Laves phase, on the contrary, drops to 10% (compared with 23% in the as-cast state). As the annealing temperature is lowered to 800 °C and then to 600 °C, the equilibrium composition of our HEA continues to change. The amount of the BCC phase A2 remains insignificant, the proportion of Laves C15 gradually drops to 60%, while the proportion of the Laves C14 phase increases to 27%. Thus, by changing the annealing temperature, one can modify the phase composition of TiZrHfMoCrCo HEAs.

HPT can significantly affect the phase composition of alloys. This happens for two reasons. The first of them is that in the stationary state during HPT, an increased concentration of defects appears in the material. Such an increased concentration of defects is in some sense equivalent to an increase in the equilibrium concentration of vacancies with increasing temperature [33]. Therefore, after HPT is stopped and the high pressure is released, the phases appear in the material, which can be found in the equilibrium phase diagram at a temperature significantly higher than the HPT temperature. In other words, HPT is equivalent to an annealing of the material at some elevated temperature followed by quenching [34]. This temperature is usually called the effective temperature *T*_eff_ [35]. The new phases appear in HEAs not only under the action of HPT, but also by other modes of severe plastic deformation such as for example ball-milling [36]. In our case, after HPT, the fraction of the C15 Laves phase remains almost constant, while the fraction of the A2 BCC phase increases from 51% to 75% due to the fact that the second C14 Laves phase completely disappears (see Table 4). Thus, the phase composition of a sample after HPT (Table 4) strongly differs from that in the samples annealed at 1000, 800, and 600 °C (Table 3). Therefore, we can suppose that the *T*_eff_ value is outside (probably above) the temperature interval of 600–1000 °C.

Annealing at 1000 °C of an alloy deformed by HPT changes again its phase composition. It resembles quite strongly the phase composition of as-cast alloy annealed at the same temperature of 1000 °C. This means that a small fraction of the BCC phase and a significant amount of Laves phases (see Table 4) indeed correspond to the equilibrium phase equilibrium of the TiZrHfMoCrCo alloy at a temperature of 1000 °C.

The second reason why HPT can affect the phase composition of materials is due to the fact that during HPT, as in other methods of severe plastic deformation, there is a significant grain refinement in the material [37]. In typical cases, the grain size decreases from hundreds of micrometers to 100–200 nanometers [38]. In this case, many new grain boundaries appear in the material [39]. Grain boundaries are characterized by the so-called grain boundary segregation. This means that the concentration of elements in a thin boundary layer differs significantly from the concentration of elements in the volume. Segregation at the boundaries can be either positive or negative. In other words, the concentration of an element at the grain boundaries can be either higher or lower than the concentration of the same element in the volume. If the grain boundary segregation is strong, then a lot of atoms are required to form grain boundary segregation layers during HPT. These segregated atoms move to the boundary from the bulk. As a result, the composition of the solid solution in the bulk can vary significantly. The solid solution are depleted in a component that strongly segregates at the boundaries. As a result, the lattice period can change so strongly that this effect becomes measurable using X-ray diffraction, as for example, in copper–silver systems or in some carbon steels [40,41]. A significant change in the lattice period after HPT and additional annealing may indicate such an effect in the TiZrHfMoCrCo HEAs studied by us (Table 4).

**Figure 11:**
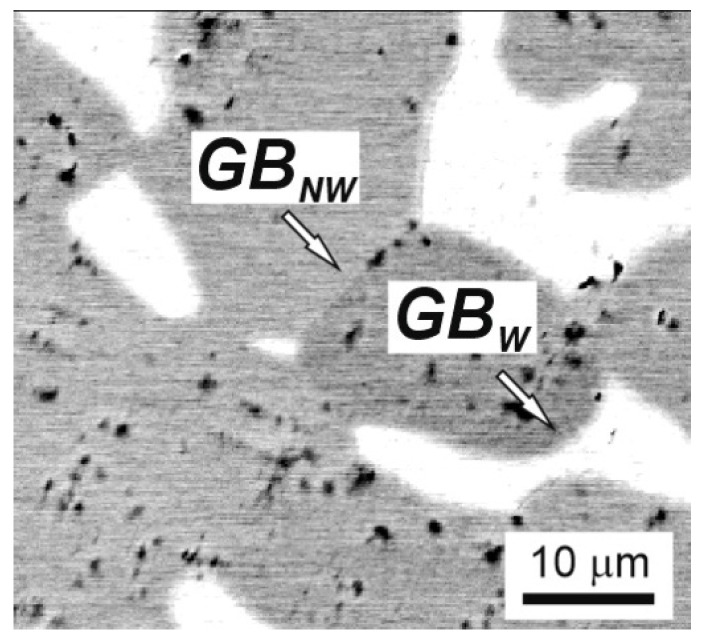
SEM micrograph of the TiZrHfMoCrCo alloy annealed at 1000 °C 336 h, where GB_W_ is the completely wetted grain boundary and GB_NW_ is the partially wetted grain boundary.

The presence of two or more phases in the alloy makes it possible to observe a phenomenon known as grain boundary wetting. In case of complete grain boundary wetting one phase substitutes the grain boundaries in another phase [17,42]. As a result, instead of one grain boundary, two interphase boundaries are formed. If one looks closely at Figure 3b, it can be seen that the grains of (C15) phase are surrounded by the layers of (A2) phase. Similar structure can be seen also in Figure 9b. It can be noticed that some (C15)/(C15) boundaries (marked as GB_W_ in Figure 11) are completely covered by the (A2) phase, and at other (C15)/(C15) boundaries (marked as GB_NW_ in Figure 11) the (A2) phase is completely absent. At a lower annealing temperature of 800 and 600 °C, the phenomenon of grain boundary wetting was not observed (see Figure 5). It means that above 1000 °C the phase transition from incomplete to complete grain boundary wetting takes place.

In most works on HEAs, the authors measured the microhardness of samples before and after the HPT [43,44,45,46,47,48,49,50]. B. Schuh et al. [45] showed that the hardness of the TiZrNbHfTa alloy sample depends not only on the annealing temperature, but also on its duration. The dependence has a nonlinear form and decreases with the duration of annealing. P.T. Hung et al. [47] confirmed the decrease in the hardness of the HfNbTiZr material when after the HPT it is subjected to annealing. H. Shahmir et al. [46] showed that the hardness of the CoCrFeNiMn alloy increases with the number of revolutions. In this work the measured values of nanohardness showed a significant difference in values for the dark (*H* = 9.5 ± 1.0 GPa) and light (*H* = 4.9 ± 0.2 GPa) phases. *H* changed almost two times. The values of the Young’s modulus also changed almost two times for the dark (*E* = 128 ± 6 GPa) and light (*E* = 232 ± 7 GPa) phases. By varying the phase ratio, it is possible to achieve the necessary characteristics of the TiZrHfMoCrCo alloy. The Laves phase has higher *H* and *E* values, which may be due to a higher cobalt content in this phase. Unfortunately, we were unable to compare the data we obtained for our alloy in terms of hardness and Young’s modulus with other authors. From the spread of values in Figure 10b, one can judge how heterogeneous the sample is along its diameter.

In this work we prepared the alloy similar to that developed in Ref. [9]. However, it turned out that the alloy prepared by us is significantly different in phase composition. There are three main phases in the alloy made by us, namely cubic (A2) one and two Laves phases (C15 and C14). In in the work of Takeshi Nagase and co-authors [9] there is one main BCC phase with a suspicion of some content of Laves phases. Since the obtained microstructures of the alloys differ, most likely the difference in the manufacturing method of the alloys was determining. One of the tasks of our work was to show the influence of the method of manufacturing of alloys on the microstructure and phase composition.

**Figure 12:**
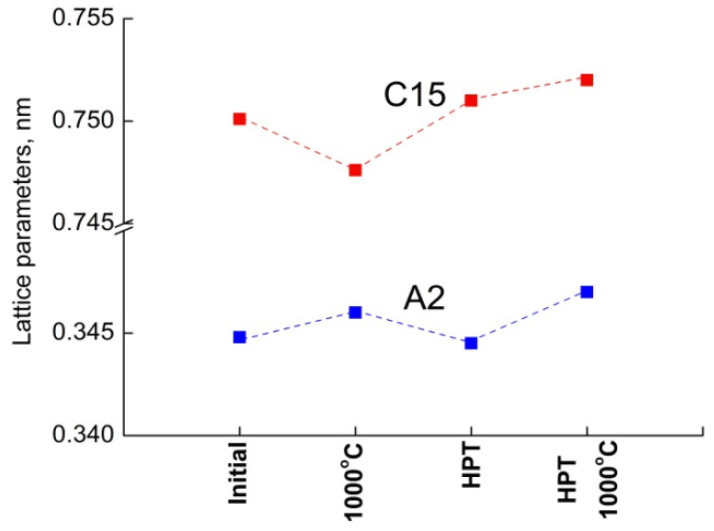
Dependence of the lattice parameter on the type of material processing of the TiZrHfMoCrCo alloy.

In the works on the HEAs, as a rule, the data on the lattice parameters of the phases are provided only seldom. The authors link the phase, grain size, hardness, and a number of other characteristics of the material. Gubicza et al. [44] (see Table 2) measured the saturation dislocation density, the grain size and the hardness for different HEAs and MEAs processed by HPT at RT. In addition to the grain size, the twin boundary (TB) spacing is also shown for some fcc HEAs with low stacking fault energy. H. Shahmir et al. [46] (see Table 1) measured the volume fraction of precipitates, grain size, microhardness of nanostructured HEA subjected to post-deformation annealing at 473–1173 K. In our work, the lattices parameters of the phases are one of the key characteristics of the material for thermal treatment and HPT exposure. All data on the lattice parameters and the phase composition of the studied samples are provided in Table 2, Table 3 and Table 4. They permit to emphasize the influence of heat treatment and HPT on the lattice parameters (Figure 12). The values of the lattice parameters were obtained by processing X-ray spectra using the PowderCell 2.4 program. In Figure 12, the designation “1000 °C” corresponds to annealing at 1000 °C for 24 h, and “HPT 1000 °C” is annealing after HPT at 1000 °C for 96 h.

As one can see, both phases react sensitively to external influences, but each phase has its own way. In the cubic (A2) phase, the heat treatment of the alloy leads to an increase in the lattice parameters, while the Laves phase (C15) is no longer predictable.

## 5. Conclusions

We investigated the effect of heat treatment and HPT on a three-phase high-entropy alloy TiZrHfMoCrCo. (i) The microstructure of the alloy under study showed good stability during these types of processing, even with HPT, it was not possible to evenly mix the phases present in the alloy. (ii) Various phase transformations in the studied alloy occur at low temperatures and HPT treatments: the additional phases such as A2-2, C14-2, and C36 appear. (iii) After HPT the grains become very small, the BCC phase prevails and C14 Laves phase completely disappears. This state is similar to the state after annealing at high effective temperature *T*_eff_. (iv) At 1000 °C the BCC phase completely wets the C15/C15 grain boundaries (GBs). At 600 and 800 °C the GB wetting is incomplete. (v) Measured values of nanohardness (A2) and (C15 + C14) of individual phases differ by two times. By selecting various combinations of heat treatment and HPT, it is possible to expand the area of phase transformations in this HEA.

## Figures and Tables

**Table 1:** The total component composition of the TiZrHfMoCrCo alloy and its individual regions (wt.%).

Point	Phase	Ti	Cr	Co	Zr	Mo	Hf
*1*	(Ti,Zr,Hf)cub (A2)	13.80 ± 0.17	0.54 ± 0.07	2.18 ± 0.90	30.00 ± 1.00	6.49 ± 0.88	46.98 ± 0.92
*2*	(Mo,Cr)_2_Zr (C15)	12.18 ± 0.47	5.28 ± 0.17	6.03 ± 1.28	16.21 ± 1.68	19.51 ± 1.10	40.85 ± 0.94
*3*	Cr_2_Zr (C14)	6.59 ± 0.12	5.21 ± 0.84	1.25 ± 0.11	11.56 ± 0.27	35.71 ± 0.27	39.59 ± 0.32
Average Composition		10.84 ± 3.26	3.82 ± 2.44	3.50 ± 3.21	18.73 ± 8.23	20.77 ± 12.99	42.34 ± 3.55

**Table 2:** Phases, lattice parameters *a*, *c* and the percentage of the phases *V* in the initial as-cast state of the TiZrHfMoCrCo alloy and after annealing at 1000 °C.

Phase	Initial Alloy		1000 °C 24 h		1000 °C 336 h	
	*a*, *c*, nm	*V*, %	*a*, *c*, nm	*V*, %	*a*, *c*, nm	*V*, %
(A2)	*a* = 0.3448	51	*a* = 0.3460	10	*a* = 0.3460	4
(C15)	*a* = 0.7501	26	*a* = 0.7476	80	*a* = 0.7473	86
(C14)	*a* = 0.5307 *c* = 0.8560	23	*a* = 0.5307 *c* = 0.8560	10	*a* = 0.5305 c = 0.8560	10

**Table 3:** Phases, lattice parameters *a*, *c*, and the percentage of the phases *V* in the TiZrHfMoCrCo alloy after anneals at 1000 °C, 800 °C, and 600 °C.

Phase	1000 °C, 336 h		800 °C, 336 h		600 °C, 480 h	
	*a*, *c*, nm	*V*, %	*a*, *c*, nm	*V*, %	*a*, *c*, nm	*V*, %
(A2)	*a* = 0.3460	4	*a* = 0.3477	3	*a* = 0.3474	3
(C15)	*a* = 0.7473	86	*a* = 0.7491	70	*a* = 0.7498	60
(C14)	*a* = 0.5305 *c* = 0.8560	10	*a* = 0.5307 *c* = 0.8560	10	*a* = 0.5310 *c* = 0.8556	27
(C14-2)	*-*	-	*a* = 0.5452 *c* = 0.8722	7	-	-
(C36)	*-*	-	*-*	-	*a* = 0.5047 *c* = 1.6368	9
Additional Phases	*-*	-	-	10	-	1

**Table 4:** Phases, lattice parameters, and the percentage of the phases *V* in the TiZrHfMoCrCo alloy after HPT and HPT plus annealing at 1000 °C.

Phase	Initial Alloy		HPT		HPT + Annealing	
	*a*, *c*, nm	*V*, %	*a*, *c*, nm	*V*, %	*a*, *c*, nm	*V*, %
(A2)	*a* = 0.3448	51	*a* = 0.3445	40	*a* = 0.3470	15
(A2-2)	*-*	-	*a* = 0.3150	35	*-*	-
(C15)	*a* = 0.7501	26	*a* = 0.7510	25	*a* = 0.7520	50
(C14)	*a* = 0.5307 *c* = 0.8560	23	-	-	*a* = 0.5348 *c* = 0.8580	30
Additional Phases	*-*	-	-	-	*-*	5

## Data Availability

The data presented in this study are available on request from the corresponding author.
